# Overview of Rheumatoid Arthritis and Scientific Understanding of the Disease

**DOI:** 10.31138/mjr.20230801.oo

**Published:** 2023-08-01

**Authors:** Mohd Jahid, Karim Ullah Khan, Rehan-Ul-Haq, Rafat Sultana Ahmed

**Affiliations:** 1 Department of Biochemistry, University College of Medical Sciences and GTB Hospital (University of Delhi), Dilshad Garden, Delhi, India,; 2 Department of Orthopaedics, University College of Medical Sciences and GTB Hospital (University of Delhi), Dilshad Garden, Delhi, India,; 3 Department of Orthopaedics, All India Institute of Medical Sciences (AIIMS) Bhopal, India

**Keywords:** rheumatoid arthritis, joints deformity, autoimmune disorder, NSAIDs and DMARDs

## Abstract

Rheumatoid arthritis (RA), a chronic inflammatory autoimmune disorder, is characterised by persistent synovial inflammation, erosion of bones and cartilage, leading to joint destruction. Clinical manifestations are morning stiffness, pain in shoulder, neck and pelvic girdle, loss of mobility with fever, fatigue, malaise, loss of body weight, and development of rheumatoid nodules. Environmental and genetic factors are important contributors in its susceptibility. Association between RA and diet, cigarette smoking, hormones, alcohol, microbiota, infection, and coffee have also been reported. To diagnose patients with RA, American college of rheumatology (ACR, 2010) criteria, developed by European league against rheumatism (EULAR). Inflammation produced in RA patients is due to cell-mediated immune response. The rheumatoid synovium consists of a large number of CD_4_^+^ T cells suggesting pathogenic nature of T cells in this disorder. B-cells may also participate in the pathogenesis by several means such as autoantibodies, by instigation of T-cells through expression of co-stimulatory molecules, by generating pro-inflammatory and anti-inflammatory cytokines and by organisation of other inflammatory cells. The conventional management of RA usually focuses over reducing pain and limiting the disability by medical therapies which include a number of classes of agents such as non-steroidal anti-inflammatory drugs (NSAIDs), non-biological and biological agents, disease-modifying anti rheumatic drugs (DMARDs), immunosuppressants, and corticosteroids. However, only proper rehabilitation can promote the objective to achieve the joint functionality and ease of motion which improves independence as well as quality of life in patient suffering from Rheumatoid Arthritis.

## INTRODUCTION

Autoimmune disorders are accountable for a large number of disabilities and morbidity which influence around 8.5% of the population worldwide. Rheumatoid arthritis (RA) is said to be a systemic chronic inflammatory disease and an autoimmune disorder.^2^ The incidence rate is 0.5% to 1% in the US population,^3^ in India 0.9%,4 while in the Middle East and North Africa it is around 0.16%.^5^ It is characterised by progressive and destructive arthropathy due to synovial inflammation and hyperplasia with increase in autoantibodies such as rheumatoid factor (RF) and anti-cyclic citrullinated peptide (anti-CCP). RA may have either of two states, active or inactive.^6^ In active state, tissue is inflamed while in inactive state the inflammation decreases.^7^ During the inactive state, patients usually do not feel any symptoms but when the disease relapses symptoms flare up.^8^ RA is a destructive symmetric polyarthritis that specifically affects the small joints^9^ leading to loss of physical functions/disability with chronic pain resulting in poor quality of life.^10^

Clinical manifestations of RA are morning stiffness, pain in shoulder, neck and pelvic girdle, loss of mobility with fever, fatigue, malaise, loss of body weight and development of rheumatoid nodules.^11–13^ Cleveland clinic Abu Dhabi reported that 43.5% of RA patients had dyslipidaemia indicating an alteration in lipid profile.^14^ Ocular engrossment is also found in 27% of RA patients including episcleritis and scleromalacia.^15^ Pulmonary involvement is frequent which includes pleural effusion, obstructive lung disorder, pulmonary vasculitis and small airway disease and is reportedly responsible for 10–20% of overall mortality in RA patients.^16,17,18^ Chronic systemic inflammation in patients with RA causes cardiovascular (CV) abnormalities beyond traditional cardiac risk factors.^19^ Hence, cardiovascular disease is a potential risk in RA patients.^20^ It is also reported that lifespan is reduced from 3–12 years in RA and risk of heart disease increases about two times.^21^ In Indian population, 7.2% small vessel vasculitis was reported in RA patients.^22^ Renal disease is also frequently observed in RA patients. Reduced kidney function with time and elevated ESR is one of the predisposing factors.^23^ In general, during treatment, it was observed that clinicians primarily focus on treating the joint manifestations and neglect to subside systemic inflammation. Thus, patients become prone to develop cardiovascular disorders.^24^ The currently used imaging techniques are echocardiography, single-photon emission computed tomography and cardiac magnetic resonance.^25^ Artificial intelligence techniques have shown promising potential for tailoring predictive medicine to the individual patients.^26^

## EPIDEMICS

**Figure 1.**
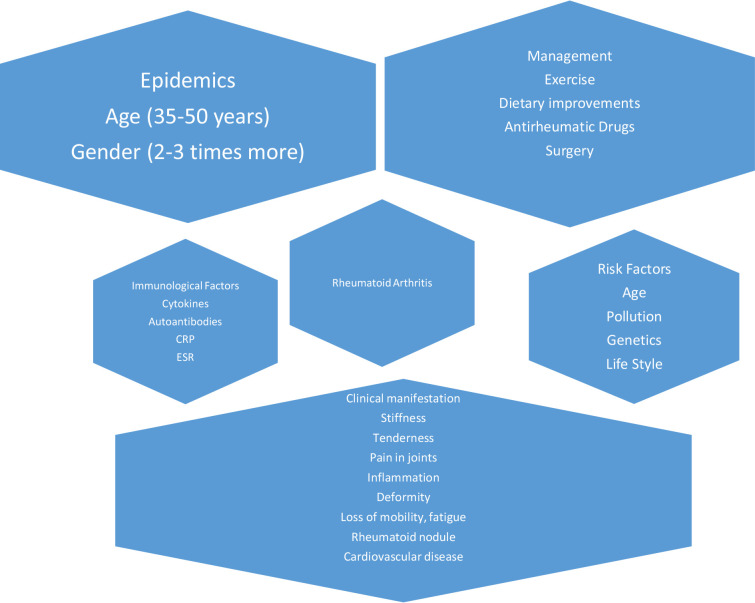
Epidemics, clinical manifestation, management, immunological factors and risk factors of rheumatoid arthritis.

Rheumatoid arthritis is a joint destructive disease, affecting people worldwide, affecting any person at any age, but often develops in fourth and fifth decade of life.^27^ Its incidence increases with increasing age.^28^ Disease onset in person above sixty-five years is called late onset while below the age of sixty it is referred to as young onset.^29^ RA is a common phenomenon in women and two to three times more frequent in comparison to men; reproductive factors and sex hormones are also considered to be involved in the aetiology of this disease.^30^ The course, prognosis and development of RA is variable and may be slow or rapid. During pregnancy, symptoms decrease in women and flare up after birth.^31^ The prevalence rate of RA in Indian population is almost similar to the incidence rate in the world. The risk of disability depends on the severity of disease onset, while disability and mortality can be increased up to 30% and 52% respectively.^32^ Risk of RA increases with positive family histories, and it is the highest contributor to worldwide disability, less than malaria and higher than iodine deficiency.^5^ Structural representation of an overview of the development of rheumatoid arthritis is given in **Figure 1**.

## COURSE OF THE DISEASE

The causes and pathophysiology of RA are not yet fully understood but it appears to be an autoimmune attack on bone and cartilage joints.^33^ RA primarily affects the membrane of synovial joints leading to disability, early death, and socioeconomic burden.^34^ It is a multifactorial disease where genetic and environmental factors play an important role in both susceptibility and onset of this ailment.^35^ In disease development there is an initial phase called as preclinical phase, during which genetic and environmental factors interact to activate a sequential phenomenon of autoimmune process which results in systemic and local inflammation of synovial joints.^36^ At the initial stage in absence of clinical signs and symptoms autoantibodies like rheumatoid factor (RF) and anti-CCP develop. At the later stage, negligible symptoms develop which may be nonspecific or uncertain for any particular rheumatic disease.^37^ The course of RA and its prognosis are inconsistent and may develop slowly or rapidly. The differences in disease progression in RA patients are because of genetic and molecular features, like patterns of inflammatory molecules present in inflammatory tissues of ailing joints.^38^

## ENVIRONMENTAL AND GENETIC FACTORS

A large number of environmental factors contribute to the development of RA. It is reported that increased urbanization is linked with an increased prevalence of this disorder.^39^ Tobacco-smoking is one of the major established environmental risk factor.^40^ Frequent RA cases have been reported in person working particularly in crop industries.^41^ Raised antinuclear antibodies (ANAs) levels were found in pest control workers involved in pesticide mixing, indicating the preclinical sign in the onset of disease.^42^ The risk of RA increases with the use of agricultural pesticides, solvents and chemical fertilizers.^43^ Many reports validate that exposure to pesticides like organophosphates, guanidine, quinone, fonofos carbaryl, and chlorimuron ethylpesticides are important risk factors for disease development.^44,45^

Association between RA and diet, cigarette smoking, hormones, alcohol, microbiota, infection and coffee have also been reported.^46–49^ Sex hormones prolactin and oestrogens predominantly found in women are also involved in the pathogenesis of RA.^50–54^ Ovarian hormones and oestradiols can control innate immune reactions by regulating cell recruitment at inflammatory tissues, spreading the responding cells and down regulating the production of pro inflammatory cytokines.^55^ In a recent study it was reported that oral contraceptives decrease the symptoms of RA, especially in anti-CCP positive patients.^56^ Association of breastfeeding with RA development has also been reported in several studies, and RA symptoms were found to be lower in breastfeeding women.^57,58^

It is also proposed that RA is an expression of the response to an agent in a genetically predisposed individual, ie, it is a result of gene-environment interaction.^49^ A potent genetic risk factor is the shared epitope (SE) alleles at HLA-DRB1.^59^ The HLA-DRB1 gene is the chief genetic susceptibility locus and its role has been confirmed by linkage and association studies of this gene.^60^ Several genetic studies reported an association of RA with certain Major histocompatibility complex (MHC) class II genes- HLA-DRB1 and HLA-DR4.^39,61,62^ The total genetic risk for RA contributes to 60% of the disease burden and HLADRB1 is likely to explain 30% to 40% of genetic risk. The non- HLA genes associated with susceptibility to this disorder are TRAF1/C5, IL2RB, AFF3, CTLA4, MMEL1, PADI4, PTPN22, TNFAIP3, STAT4, CD6, CD40, KIF5A-PIP4K2C and CCL21.^63^ The widely employed approach for the study of susceptibility variants in rheumatoid arthritis is genome wide association studies (GWAS). GWAS has helped in the identification and validation of several novel RA risk alleles. The discovery of rare variations is still challenging in the current scenario of genotyping arrays. In the present paradigm, it is assumed that RA is triggered in genetically predisposed individuals by exposure to environmental factors, and may be associated with epigenetic changes.

## DIAGNOSIS

American College of Rheumatology classification criteria (1987) was formerly used to enrol the patients with RA, but due to lack of sensitivity in early RA, it was criticised and replaced by the American College of Rheumatology (ACR, 2010) criteria, developed by European League Against Rheumatism (EULAR).^64^ Different scoring systems are used to quantify the RA disease activity but mostly disease activity score 28 (DAS 28) is used. This scoring system is based on 28 counts of tender and swollen joints.^65^ Erythrocyte sedimentation rate (ESR) and C-reactive protein (CRP) are the acute phase reactants. ESR is used to measure the level of inflammation and reflects the increased concentration of acute phase plasma proteins. CRP is an established inflammatory marker in RA, and is synthesised by the hepatocytes in response to stimulation by proinflammatory cytokines.^66,67^ Imaging is the most common non- invasive technique and traditional primary tool to monitor the disease progression and severity. Radiographs of hands and feet are used to study the progression, wherein the degree of bone erosion and narrow space in joints represents the loss of cartilage. There is an association among Doppler ultrasound and MRI oedema with erosive radiographic progression of disease.^68^ Scintigraphy plays a crucial role in the differential diagnosis of hip and knee joint effusion in patients with RA.^69^

## PATHOGENESIS

A large number of diverse cellular responses are concerned with the pathogenesis of this disorder, which includes activation of inflammatory cells, expression of different cytokines, local growth factors, and local angiogenesis. T-cells, B-cells, neutrophils, and macrophages are mainly present in synovial tissue and generate inflammatory as well as degradative molecules that break down the extracellular matrix (ECM) of cartilages and bones.^70^ Inflammation produced in RA patients is due to cell mediated immune response. The rheumatoid synovium consists of a large number of CD_4_^+^ T cells and activation of CD_4_^+^ T cells is considered to be antigen driven, signifying a pathogenic function for T cells in this joint disease.^71^ It is proposed that persistent inflammation in RA is due to the interactions among T-cells, macrophages, and fibroblasts.^72^ Breach of self-tolerance and activation of naive antigen specific T cells by antigen presenting cells particularly by dendritic cells is a crucial step in the development of autoimmune disorders.^73^

B-cells may participate in the pathogenesis of rheumatoid arthritis by several means such as producing autoantibodies and instigation of T-cells by expression of co-stimulatory molecules, generation of proinflammatory and anti-inflammatory cytokines and the organisation of other inflammatory cells.^74^ The inflamed joints exhibit severe synovitis and erosion of the adjacent cartilages and bones causing articular destruction.^59^ One of the causes of tissue damage is the plethora of pro-inflammatory cytokines secreted by neutrophils.^75^ The connective tissue is destructed by specific kind of protein degrading enzymes- Matrix metalloproteinases (MMPs) which may degrade proteins of the ECM and frequently require definite stimuli to upregulate production. One of the most copious MMPs found in synovium and synovial fluids in patients with RA is MMP-3.^76^

Secretion of cytokines from activated T-cells and B-cells induces proliferation and activation of the synovial and epidermal fibroblasts leading to the development of clinical symptoms in patients.^77^ Serum cytokines are known to play a crucial role in the pathogenesis by initiating and perpetuating various humoral and cellular autoimmune components of the immune system and are usually generated in a cascade, as a particular cytokine induces the target cells to produce, in turn, the other cytokines. ^73^ Pro-inflammatory cytokines, TNF-α and IL-1β stimulate synovial fibroblasts, osteoclasts, macrophages, and chondrocytes. These synovial cells produce ECM destroying matrix metalloproteinases (MMPs), particularly MMP-1 and MMP-3 which participate in tissue destruction events such as cartilage degradation.^78^

TNF-α is considered an autocrine stimulator and a paracrine inducer of IL-1 and granulocyte macrophage colony stimulating factor (GM-CSF). Thus, TNF-α heightens its production by the positive feedback of its own gene expression. TNF-α is known to contribute to joint inflammation associated with RA, as illustrated by the effects of neutralising TNF-α to ameliorate inflammation.^79^ IL-1 is one of the most potent pro-inflammatory cytokines and plays a pivotal role in inflammation and destruction of joint bones and cartilages. It stimulates biosynthesis of IL-6, IL-8 and GM-CSF and subsequently induces the expression of adhesion molecules such as VCAM-1 and ICAM-1.^73^ Interleukin-10 (IL-10) is a pleiotropic cytokine that stimulates B-cell survival, proliferation, differentiation, and antibody isotype switching and has an important role in the pathogenesis of this disorder.^80^ The levels of TNF-α, IL-1β, and IL-10 are found to be elevated in serum of the patients. The polymorphisms of cytokine genes are potentially significant as genetic predictors of the disease susceptibility or clinical outcome because the gene products of these cytokines are involved in the pathogenesis of this disease.^81–83^ Severity depends upon difference in the levels of cytokine production.

Oxidative stress and antioxidants also play a key role in the pathogenesis of RA.^84^ Equilibrium between reactive oxygen species (ROS) formation and antioxidant system of the cell is disrupted due to oxidative stress and as a result there is damage of vital cell components such as proteins, DNA and membrane lipids. Formation of ROS results in oxidation of DNA and lipids giving rise to a variety of cytotoxic products such as lipid and DNA hydroperoxides and alkanals.^85^ Lipid peroxidation is reported to be significantly higher and nonenzymatic anti-oxidant vitamin C is significantly lower in patients with rheumatoid arthritis.^86^

## TREATMENT STRATEGIES

To this day, there is no permanent cure available for this disease. Treatment is aimed at slowing the progression of the disease, diminish inflammation and pain, retain joint functions to minimise joint damage and complications as well as enhancing physical function and quality of life. Best possible care for patients with RA includes an integrated approach that includes non-pharmacologic as well as pharmacologic therapeutic interventions. Physical activity is an important intervention for improving systemic manifestations in rheumatic and musculoskeletal disease (RMDs).^87^ Many non-pharmacologic treatments are available for this ailing disease, including tailored exercise,^87^ specifically endurance exercise, diet, occupational therapy, stress reduction, physiotherapy, and surgery.^88^ Exercise intervention can significantly improve disease outcome comprising FITT (frequency, intensity, time, and type) as well as training (specificity, overload, progression, reversibility, and diminishing return).^89^ It improves the cardiorespiratory fitness and disease activity in RA patients with a risk for CVD.^90,91^ Psychotherapy helps to improve self-confidence and regaining positive attitude to cope with the depression and associated comorbidities in patients.^92^

The conventional management of RA focuses over reducing pain and limiting the disability, which involves therapies based on medication and includes a number of classes of agents such as non-steroidal anti-inflammatory drugs (NSAIDs), non-biological and biological agents, disease modifying anti rheumatic drugs (DMARDs), immunosuppressants, and corticosteroids. NSAIDs are commonly used as first-line agents for the symptomatic improvement of pain, swelling and morning stiffness in several inflammatory conditions. NSAIDs-Ibuprofen, Diclofenac, Naproxen, Celecoxib, and Meloxicam are anti-inflammatory and analgesic drugs.^8^ DMARDs are said to alter the disease course, retard progression, reduce the activity of disease, and restore radiographic outcomes. Therefore, DMARDs have been established as the standard of care in allopathic system for the treatment.^93^ The currently available drugs include methotrexate (MTX), hydroxychloroquine, sulfasalazine, sulphapyridine and leflunomide. MTX is a favourite choice among physicians and is now considered as the first-line DMARD agent for treatment of RA, due to ease of administration, relatively low cost, quick onset of action at therapeutic doses, and efficacy.^94,95^

Biological agents are used to treat moderate to severe RA, especially in patients who do not respond effectively to other treatments and have poor prognosis. Biologics help to slow down progression of RA when all other treatments have failed. These may be used alone but are often given in combination with other medications such as NSAIDs. Use of TNF-α inhibitors have revolutionised the era of RA treatment options resulting in the expansion of further biological DMARDs.^96^ Anti TNF-α drugs such as infliximab (IFX), etanercept (ETN), adalimumab (ADA), golimumab (GOLI), and certolizumab pegol (CZP) have been extensively used as treatment options.^97^ Canakinumab is one such anti-IL-1β drug developed to cure the disease.^98,99^ Several other anti-interleukin monoclonal antibodies used in the management of RA are, Olokizumab, Sirukumab, Tocilizumab, Sarilumab, Briakinumab, Ixekizumab.^100^ Over-the-counter (OTC) medications, complementary, and alternative treatments such as herbal and indigenous medicines can also be helpful to relieve pain and reduce inflammation in RA patients.

## REHABILITATION OF PATIENTS

Rehabilitation of RA patients is a subject of prime importance and is aimed to improve or maintain the life of RA patients. Rehabilitation involves the methods and techniques such as tailored exercise,^87^ for the correction and restoration of normal functions, minimize the complications in work, restoration and preservation of the work capacity in RA patients.^101^ The role of occupational therapists in rehabilitation process is vital which helps patients to manage and retain their normal functional condition.^102^

## CONCLUSION

Rheumatoid Arthritis is a chronic inflammatory disease characterised by progressive, symmetric joint inflammation and subsequent deformity. RA is caused by imbalance between pro- and anti-inflammatory cytokines which promotes inflammation, oxidative stress, and joint destruction. Treatment strategies include steroids, DMARDs, and biological therapies. However, only proper rehabilitation can promote the objective to achieve the joint functionality and ease of motion which improves independence as well as quality of life in patients suffering from Rheumatoid Arthritis.

## ABBREVIATION LIST

ADA: Adalimumab
ACR: American College of Rheumatology
anti-CCP: Anti-cyclic citrullinated peptide
ANAs: Antinuclear antibodies
CRP: C-reactive protein
CV: Cardiovascular
CZP: Certolizumab pegol
DAS 28: Disease activity score 28
DMARDs: Disease-modifying anti rheumatic drugs
ESR: Erythrocyte sedimentation rate
ETN: Etanercept
EULAR: European League Against Rheumatism
ECM: Extracellular matrix
GWAS: Genome wide association studies
GOLI: Golimumab
GM-CSF: Granulocyte macrophage colony stimulating factor
IFX: Infliximab
IL-10: Interleukin-10
MHC: Major histocompatibility complex
MMPs: Matrix metalloproteinases
MTX: Methotrexate
NSAIDs: Non-steroidal anti-inflammatory drugs
OTC: Over-the-counter
ROS: Reactive oxygen species
RA: Rheumatoid arthritis
RF: Rheumatoid factor
SE: Shared epitope
